# The neuropsychological assessment battery (NAB) is a valuable tool for evaluating neuropsychological outcome after aneurysmatic subarachnoid hemorrhage

**DOI:** 10.1186/s12883-020-02003-9

**Published:** 2020-11-26

**Authors:** Johannes Walter, Martin Grutza, Lidia Vogt, Andreas Unterberg, Klaus Zweckberger

**Affiliations:** Department of Neurosurgery, University of Heidelberg, Im Neuenheimer Feld 400, 69120 Heidelberg, Germany

**Keywords:** Subarachnoid hemorrhage, Aneurysmatic subarachnoid hemorrhage, Hemorrhagic stroke, Aneurysm, Neuropsychology, Neuropsychological assessment battery

## Abstract

**Background:**

Detecting and treating neuropsychological deficits after aneurysmatic subarachnoid hemorrhage (aSAH) play a key role in regaining independence; however, detecting deficits relevant to social and professional reintegration has been difficult and optimal timing of assessments remains unclear. Therefore, we evaluated the feasibility of administering the Neuropsychological Assessment Battery screening module (NAB-S) to patients with aSAH, assessed its value in predicting the ability to return to work and characterized clinical as well as neuropsychological recovery over the period of 24 months.

**Methods:**

A total of 104 consecutive patients treated for aSAH were recruited. After acute treatment, follow up visits were conducted at 3, 12 and 24 months after the hemorrhage. NAB-S, Montreal Cognitive Assessment (MoCA) and physical examination were performed at each follow up visit.

**Results:**

The NAB-S could be administered to 64.9, 75.9 and 88.9% of the patients at 3, 12 and 24 months, respectively. Moderate impairment of two or more neuropsychological domains (e.g speech, executive function, etc.) significantly correlated with inability to return to work at 12 and 24 months as well as poor outcome assessed by the extended Glasgow Outcome Scale (GOSE) at 3, 12 and 24 months. The number of patients with favorable outcomes significantly increased from 25.5% at discharge to 56.5 and 57.1% at 3 and 12 months, respectively, and further increased to 74.1% after 24 months.

**Conclusion:**

The NAB-S can be administered to the majority of patients with aSAH and can effectively detect clinically relevant neuropsychological deficits. Clinical recovery after aSAH continues for at least 24 months after the hemorrhage which should be considered in the design of future clinical trials.

## Background

Aneurysmatic subarachnoid hemorrhage (aSAH) is caused by rupture of an intracranial aneurysm and leads to significant long-term morbidity. As many cases of aSAH occur at a relatively young age with peak incidence at 55 years of age, many survivors are faced with limitations in their everyday life for decades [1–4]. Besides physical impairments, neuropsychological deficits have been recognized as one of the main factors affecting patient’s quality of life; however, even though many outcome assessment tools like the Montreal Cognitive Assessment (MoCA) and the Mini-Mental Status Evaluation (MMSE), different memory tests like the Wechsler Memory Scale or the California Verbal Learning Test as well as tests of executive function, such as the Wisconsin Card Sorting Task, have been evaluated, it has been difficult to recognize subtle cognitive deficits by means of gross neurological measures [5–10]. Therefore, it is the goal of the current study to evaluate the feasibility of administering the Neuropsychological Assessment Battery screening module (NAB-S) to patients with aSAH. The NAB is a scale specifically developed to neuropsychologically evaluate neurologically impaired patients. It consists of a screening module which is used in the current study and five additional modules that evaluate the five domains screened with the screening module in more detail. Unlike other tests used to evaluate distinct neurocognitive domains like executive or memory function that only provide information on one single domain, the NAB-S provides information on five different domains (attention, language, memory, spatial und executive function) in a single test battery. On the other hand, it provides more detailed information than other combined test batteries that also evaluate multiple domains like the MMSE or the MoCA. Therefore, the NAB-S is a highly interesting new tool for evaluating multiple domains of neuropsychological function in a short period of time with only one single test battery.

In addition to evaluating the feasibility of applying the NAB-S to patients with aSAH, we aimed at characterizing the course of clinical and neuropsychological recovery within the first 2 years after aSAH. Many large clinical studies only evaluated outcome at 3 months after aSAH; however, significant recovery might continue for at least 24 months [4, 11–16]. Therefore, in our study patients were followed up for at least 24 months in order to develop a clinically significant follow up plan that might influence future clinical study design.

## Methods

### Patients

After approval of the protocol by the local standing committee on ethical practice of the University of Heidelberg (Ethics Committee of the Medical Faculty, University of Heidelberg, Heidelberg, Germany, Approval Number S-642/2018), a total of 104 consecutive patients treated for aneurysmatic subarachnoid hemorrhage at our academic neurosurgical clinic, which is part of a large university hospital, were prospectively recruited after providing written informed consent in accordance with the Declaration of Helsinki. If their clinical condition hampered patients from providing written consent themselves it was obtained from legal proxies. All patients were treated on the neurosurgical intensive care unit according to local standard of care consisting of rapid diagnostics including native computed tomography (CT), CT angiography and digital subtraction angiography (DSA) as well as securing the ruptured aneurysm by either clipping or coiling after interdisciplinary case discussion and, if necessary, diversion of cerebrospinal fluid (CSF) followed by neurointensive care treatment. All patients received physiotherapy while being hospitalized in our institution and were referred to postacute neurorehabilitation thereafter.

### Follow up

Follow up consultations were scheduled 3, 12 and 24 months after the hemorrhage. As 20 patients died during the acute phase on the intensive care unit (ICU) and four more patients had not reached the first follow time point at the time of data analysis a maximum of 80 patients was available for neurological and neuropsychological outcome assessment. Prior to follow up patients or their legal proxies were contacted by phone to evaluate whether extensive neuropsychological examination was possible. If patients were able to follow simple commands they were scheduled for clinical and neuropsychological follow up. Otherwise a structured telephone interview consisting of the standardized GOSE telephone interview based on the suggestions published by Wilson et al. in 1998 and additional medical questions was conducted [17]. At follow up visits clinical examination including a thorough neurological assessment as well as administration of the MoCA test and the German version of the NAB-S were performed. Neurological assessments were conducted by a physician while neuropsychological assessments were conducted by a clinical neuropsychologist, who was blinded to the patients` treatment and clinical course at all follow-up visits.

### Montreal cognitive assessment (MoCA)

The MoCA is a cognitive screening tool that was originally developed in 2005 to assist physicians in diagnosing mild cognitive impairment [18]. It consists of 13 tasks and evaluates multiple domains of cognitive function (visuospatial abilities, attention, concentration, language, short-term and working memory). It can be administered within 15 min and has been used to screen patients with a wide range of conditions including aSAH [19–22]. The maximum score is 30 points and using a cutoff of 25 points is suggested to detect mild cognitive impairment; therefore, in the current study a MoCA score of 25 or less was categorized as impaired.

### Neuropsychological assessment battery (NAB)

The Neuropsychological Assessment Battery is a comprehensive, modular test battery which was developed to evaluate neuropsychological function in neurologically impaired patients [23]. The initial standard random sample consisted of 1.448 individuals aged 18 to 97. As age-, education- and gender-corrected norms were developed, it can be applied to a wide range of patients. The NAB consists of six modules: A screening module briefly evaluating each of the five domains attention, language, memory, spatial und executive function, as well as five additional modules each of which evaluates one of the domains in more detail. Different modules can be combined to create an individual neuropsychological assessment tailored to the patient’s deficits and span of attention. In the current study, the German version of the NAB screening module (NAB-S) was used [24–26]. It can be applied within 45 min which is an appropriate timeframe for patients who have suffered from aSAH and rendered feasible in a clinical setting. In contrast to many tests that have previously been used to assess neuropsychological function in patients with aSAH, like the Wechsler Memory Scale or the California Verbal Learning Test, which only assess one neurocognitive domain, the NAB-S provides multidimensional information on neuropsychological function [26–28]. In addition, it provides more detailed information than other screening tests like the MoCA or the MMSE combining detailed information on all domains of neuropsychological function despite being a screening test that can be applied within a short period of time [29]. Finally, the NAB-S is well suited for repeat testing as two parallel test versions are available making it suitable for long term outcome assessments.

In the current study, the NAB-S was administered by a clinical neuropsychologist at 3,12 and 24 months after the hemorrhage. The patient’s performance was compared to age-, education-, and gender-corrected norms and index values were determined for each tested domain. Finally, the results were stratified into six categories according to the index values: severe impairment, moderate impairment, mild impairment, average performance, performance above average and exceptional performance. Mild impairment was present if the patient’s index value was within one standard deviation below average while test results were classified as moderately impaired if index values were between one and two standard deviations below average. If patients scored more than two standard deviations below average their performance was classified as severely impaired. Results were stratified as above average if scores were within one standard deviation above average and an exceptional performance was present if patients scored more than one standard deviation above average.

### Statistical analysis

Continuous variables were compared using one way ANOVA for normally distributed variables or Mann–Whitney-U-Test for nonnormally distributed variables. Discrete variables were compared using Chi^2^ or Fisher’s exact test when appropriate. Statistical significance was defined as *p* ≤ 0.05.

## Results

### Baseline characteristics

Baseline characteristics are shown in Table 1. Mean age was 56.4 +/− 13.7 years and 43.3% of the patients were female. 56.7% of the patients had low grade aSAH (WFNS grades I-III) while 43.3% of the patients suffered from poor grade aSAH (WFNS grades IV and V). In 90.7% of the cases the source of bleeding was located in the anterior circulation while 9.3% of the ruptured aneurysms were located in the posterior circulation. The imbalance in favor of anterior circulation aneurysms is due to the fact that in our facility patients with aSAH are treated on both the neurosurgical and the neurological ICU; however, patients with aneurysms more likely to be clipped which are aneurysms of the anterior circulation in most cases are preferentially treated at the neurosurgical ICU. Mean aneurysm size was 6.7 +/− 3.8 mm and significantly higher in poor grade aSAH compared to low grade aSAH (8.0 +/− 4.4 mm vs. 5.8 +/− 3.1 mm, *p* = 0.009).

**Table 1:** Baseline characteristics of the enrolled patients

	Total	WFNS I-III	WFNS IV + V	*p*-value
Number	100% (104/104)	56.7% (59/104)	43.3% (45/104)	
Age (years)	56.4 +/− 13.7	57.8 +/− 15.2	54.5 +/− 11.3	0.22
Female sex	43.3% (45/104)	47.5% (28/59)	37.8% (17/45)	0.43
Male sex	56.7% (59/104)	52.5% (31/59)	61.2% (28/45)	0.43
Anterior circulation aneurysm^a^	90.7% (97/107)	93.5% (58/62)	86.7% (39/45)	0.43
Posterior circulation aneurysm^a^	9.3% (10/107)	6.5% (4/62)	13.3% (6/45)	0.43
Mean aneurysm size (mm)	6.7 +/− 3.8	5.8 +/− 3.1	8.0 +/− 4.4	**0.009**

^a^*In three cases of low grade aSAH more than one aneurysm was detected by initial angiography*

### Treatment and ICU stay

In 8.4% of the patients the aneurysm was not treated due to poor clinical prognosis. In three cases two aneurysms were diagnosed and consecutively treated in the same patient. The aneurysms were treated by clipping in 42.9% and by coiling in 57.1% of the cases. Mean length of stay on the ICU or intermediate care unit (IMC) was 21.2 +/− 10.5 days. Patients with low grade aSAH were treated on the ICU or IMC for a significantly shorter period of time than patients suffering from poor grade aSAH (18.9 +/− 10.3 days vs. 24.9 +/− 9.7 days, *p* < 0.001). During ICU treatment 56.7% of the patients developed delayed cerebral ischemia (DCI) which was defined as new focal neurological deficit persisting for more than 1 h, drop of oxygen pressure in the cerebral tissue (pTiO2) below 15 mmHg determined by intraparenchymal pTiO2-probe, acceleration of mean velocity in the middle cerebral artery (MCA) above 160 cm/s determined by transcranial doppler sonography, cerebral perfusion deficit determined by CT angiography or cerebral vasospasm diagnosed by DSA. All cases of DCI were treated with elevation of mean arterial pressure (MAP); furthermore, in 76.6% of the patients with DCI intraarterial Nimodipin was administered and percutaneous balloon angioplasty for treatment of macro-vasospasm was performed in 4.3% of the cases. Decompressive hemicraniectomy for refractory elevated intracranial pressure was performed in 19.4% of the cases. Total in hospital mortality was 19.2% dropping to 10.6% when only accounting for cases with initial treatment of the ruptured aneurysm. In hospital mortality in low grade aSAH was significantly lower than in poor grade aSAH (8.5% vs. 33.3%, *p* = 0.003). Median Glasgow Coma Score (GCS) at discharge was 13.0. Median Extended Glasgow Outcome Scale (GOSE) score at discharge was 3.0 with 25.5% of the patients reaching a favorable outcome of GOSE score five or better and 29.5% of the patients scoring 3 or better on the modified Rankin Scale (mRS). In low grade SAH, median GCS and GOSE scores at discharge were 15 and 4.5, respectively, with 47.1% of the patients reaching a GOSE score of five or more and 50.0% scoring 3 or better on the mRS. In poor grade SAH, median GCS and mean GOSE scores at discharge were 9.0 and 2.0, respectively. No patient suffering from poor grade SAH reached a GOSE score of five or more and only 4.7% scored 3 or better on the mRS at discharge, indicating a significantly worse outcome at discharge compared to low grade aSAH (*p* < 0.001 for both comparisons, Table 2).

**Table 2:** Treatment characteristics and in-hospital outcome. WFNS: World Federation of Neurological Surgery, DCI: Delayed cerebral ischemia, ICU: Intensive care unit, GCS: Glasgow Coma Score, GOSE: Glasgow Outcome Score Extended, mRS: Modified Rankin Scale

	Total	WFNS I-III	WFNS IV + V	*p*-value
Clipping^a^	42.9% (42/98)	39.3% (24/61)	48.6% (18/37)	0.49
Coiling^a^	57.1% (56/98)	60.6% (37/61)	51.4% (19/37)	0.49
No Treatment^b^	8.4% (9/107)	1.6% (1/62)	17.8% (8/45)	**0.009**
DCI	56.7% (47/83)	49.1% (26/53)	70.0% (21/30)	0.11
Intraarterial Nimodipine for DCI	76.6% (36/47)	73.1% (19/26)	81.0% (17/21)	0.73
Balloon Angioplasty for DCI	4.3% (2/47)	7.7% (2/26)	0% (0/21)	0.50
Decompressive Hemicraniectomy	19.4% (19/98)	11.5% (7/61)	32.4% (12/37)	**0.02**
Mean ICU stay (days)	21.2 +/− 10.5	18.9 +/− 10.3	24.9 +/− 9.7	**< 0.001**
In-hospital mortality	19.2% (20/104)	8.5% (5/59)	33.3% (15/45)	**0.003**
GCS at discharge (median)	13.0 (3–15)	15.0 (3–15)	9.0 (3–15)	
GOSE at discharge (median)	3.0 (1–8)	4.5 (1–8)	2.0 (1–4)	
mRS at discharge (median)	5.0 (0–6)	3.0 (0–6)	5.0 (3–6)	
GOSE ≥5	25.5% (24/94)	47.1% (24/51)	0% (0/43)	**< 0.001**
mRS ≤3	29.5% (28/95)	50.0% (26/52)	4.7% (2/43)	**< 0.001**

^a^*In three cases of low grade aSAH two aneurysms were treated within the initial ICU stay because the source of bleeding could not be determined with certainty in two cases an in one case a second aneurysm was located in close proximity to the ruptured one*

^b^*In one case of low grade aSAH treatment was intentionally delayed because of presence of severe vasospasm on initial angiogram (the patient was admitted 8 days after the hemorrhage). The patient died due to fulminant pulmonary embolism 10 days later before the aneurysm had been secured*

### Clinical outcome at follow up

The number of patients with a GOSE score of five or better indicating independence at home but inability to return to work significantly increased to 56.5 and 57.1% at 3 and 12 months, respectively (*p* < 0.001 and *p* = 0.002, respectively) indicating significant neurological improvement within the first 12 months after the hemorrhage. Between 12 and 24 months, the number of patients with a GOSE score of five or more further increased to 74.1% indicating a trend towards further functional recovery in the second year after the bleeding. Patients with low grade aSAH were significantly more likely to reach a GOSE of 5 or better after 3 months compared to patients with poor grade aSAH (77.8% vs. 26.3%, *p* = 0.002). At 12 months this difference could also be detected (73.7% vs. 37.5%, *p* = 0.07).

There was a significant increase in number of patients with an mRS score of three or less indicating the ability to walk without assistance but requiring some help with daily activities at 3 and 12 months compared to discharge (69.6 and 68.6% vs. 29.5%, *p* < 0.001 for both comparisons) indicating significant neurological improvement within the first 12 months after bleeding. Between 12 and 24 months, the number of patients with a mRS score of three or more further increased to 77.8%. At 3 months the number of patients with a mRS of 3 or better was significantly higher in low grade aSAH compared to poor grade aSAH (85.2% vs. 47.4%, *p* = 0.016). This difference was reduced at 12 and 24 months (78.9% vs. 56.3 and 87.5% vs. 63.6%, respectively, Table 3).

**Table 3:** Outcome at follow up consultations. A GOSE of five indicates independence at home but inability to return to work while a mRS of three indicates the ability to walk without assistance but requiring some help with daily activities. GOSE: Glasgow Outcome Score Extended, mRS: Modified Rankin Scale, NAB-S: Neuropsychological Assessment Battery screening module, MoCA: Montreal Cognitive Assessment

	3 Months				12 Months				24 Months			
	Total	WFNS I-III	WFNS IV + V	***p***-value	Total	WFNS I-III	WFNS IV + V	***p***-value	Total	WFNS I-III	WFNS IV + V	***p***-value
GOSE ≥5	56.5% (26/46)	77.8% (21/27)	26.3% (5/19)	**0.002**	57.1% (20/35)	73.7% (14/19)	37.5% (6/16)	0.07	74.1% (20/27)	81.3% (13/16)	63.6% (7/11)	0.39
mRS ≤3	69.6% (32/46)	85.2% (23/27)	47.4% (9/19)	**0.016**	68.6% (24/35)	78.9% (15/19)	56.3% (9/16)	0.28	77.8% (21/27)	87.5% (14/16)	63.6% (7/11)	0.19
NAB-S impossible	35.1% (13/37)	23.8% (5/21)	50.0% (8/16)	0.19	24.1% (7/29)	15.4% (2/13)	31.3% (5/16)	0.41	11.1% (3/27)	6.3% (1/16)	18.2% (2/11)	0.55
Impaired MoCA	68.6% (24/35)	60.0% (12/20)	80.0% (12/15)	0.28	66.7% (18/27)	50.0% (6/12)	80.0% (12/15)	0.13	58.3% (14/24)	64.3% (9/14)	50.0% (5/10)	0.68
Returned to work	0% (0/42)	0% (0/23)	0% (0/19)	1.0	23.5% (8/34)	36.8% (7/19)	6.7% (1/15)	**0.05**	37.5% (9/24)	53.8% (7/13)	18.2% (2/11)	0.11

### Neuropsychological outcome

NAB screening was not possible due to clinical condition in 35.1% of the patients at 3 months. The number of patients in which NAB screening was impossible due to clinical condition decreased throughout the first 24 months (24.1 and 11.1% at 12 and 24 months, respectively).

If medium or more severe impairments were present in two or more domains of the NAB-S significantly more patients had a GOSE score of 4 or less compared to patients without these impairments at 3, 12 and 24 months (66.7% vs. 0%, *p* = 0.003; 66.7% vs. 7.7%, *p* = 0.017 and 75.0% vs. 0%, p = 0.003, respectively).

Patients with intraparenchymal or intraventricular blood clots (Fisher °IV) were more likely to have two or more moderately impaired domains of the NAB at 12 months compared to patients with subarachnoid blood clots only (Fisher °I-III; 44.4% vs. 0%), however, this difference leveled off by 24 months (30.8% vs. 20.0%).

Patients with DCI were more likely to have moderate impairment of at least two domains of the NAB-S at 24 months (50.0% vs. 12.5%), however, this difference did not reach statistical significance due to small number of patients. There was no difference between the two groups at 12 months (36.4% vs. 30.0%).

At 3 and 12 months, 31.4 and 33.3% of the patients scored 26 or more points in the MoCA and therefore had an unimpaired MoCA score. The number of patients with unimpaired MoCA scores increased to 41.7% at 24 months.

### Return to work

Data on occupational status is summarized in Table 3**.** No patient was able to return to work at 3 months. After 12 months, 23.5% of the patients had returned to work indicating a significant increase (*p* < 0.001). After 24 months the number of patients who had returned to work further increased to 37.5%. Low grade aSAH was associated with significantly higher numbers of patients who had returned to work at 12 months compared to poor grade aSAH (36.8% vs. 6.7%, *p* = 0.05). This trend persisted at 24 months (53.8% vs 18.2%).

There was no severe impairment of any domain of the NAB-S in any of the patients who had been able to return to work at any timepoint after the hemorrhage. If no severe impairment was present in any domain of the NAB-S significantly more patients were able to return to work at 12 and 24 months compared to patients with severe impairment of any NAB-S domain (0% vs. 50.0%, *p* = 0.047 and 0% vs. 57.1%, *p* = 0.045, respectively).

No patient with at least medium impairment of two or more NAB-S domains was able to return back to work at either 3, 12 or 24 months. Significantly less patients were able to return to work at 12 and 24 months if medium impairment of two or more NAB-S domains were present (0% vs. 50.0%, *p* = 0.047 and 0% vs. 57.1%, *p* = 0.045, respectively).

At 12 months, one patient with an impaired MoCA score of 25 points and at 24 months two patients with impaired MoCA tests were able to return to work. Patients were more likely to be able to return to work at 12 months if they had scored 26 points or more compared to patients who scored less than 26 points (57.1% vs. 14.3%); however, this difference was not statistically significant due to small patient number. The number of unimpaired MoCA scores in the group of patients who had been able to return to work was not different from the group of patients who had not been able to return to work at 24 months (40.0% vs. 22.2%). Table 4 summarizes the correlation between neuropsychological outcome and the ability to return to work.

**Table 4:** Correlation between neuropsychological outcome and the ability to return to work. The NAB-S assesses the five domains attention, language, memory, spatial und executive function. MoCA was considered impaired when scoring less than 26 points. NAB-S: Neuropsychological Assessment Battery screening module, MoCA: Montreal Cognitive Assessment

		Medium impairment of NAB-S domains			Severe impairment of NAB-S domains			MoCA		
		< 2	≥2	*p*-value	< 1	≥1	*p*-value	impaired	unimpaired	*p*-value
**Return to work**	12 months	50.0% (7/14)	0% (0/7)	**0.047**	50.0% (7/14)	0% (0/7)	**0.047**	14.3% (1/7)	57.1% (4/7)	0.266
	24 months	57.1% (8/14)	0% (0/5)	**0.045**	57.1%(8/14)	0% (0/5)	**0.045**	22.2% (2/9)	40.0% (2/5)	0.580

## Discussion

### Clinical outcome

It has been noted that neuropsychological deficits have a significant impact on patients` recovery from aSAH [30]. In our study, moderate impairment of neuropsychological function was also significantly correlated to poor outcome assessed by GOSE at 3, 12 and 24 months after the hemorrhage. On one hand, this underscores the importance of neuropsychological assessment as part of follow up schedules for patients with aSAH to detect deficits that might be amenable to therapeutic intervention. On the other hand, we could show that the NAB-S can effectively detect neuropsychological deficits relevant for recovery in a cohort of patients with aSAH, and therefore is a promising tool for implementation in SAH follow up strategies.

The presence of intraparenchymal or intraventricular blood clots on initial CT imaging was associated with moderate impairment of at least two domains of the NAB-S at 12 months in our study. This is well in line with the results of previous studies indicating a relationship between the initial Fisher score and neuropsychological deficits and highlights the importance of blood location and clot size for neuropsychological outcome [31–35]. This might be due to the relation of Fisher grade and development of delayed cerebral ischemia; however, in our study, the development of DCI did not significantly correlate with more severe neuropsychological impairment indicating an important role of parenchymal lesions due to initial intraparenchymal hemorrhage and chronic hydrocephalus in the development of neuropsychological deficits within the first 12 months after the bleeding. However, as more patients with DCI developed neuropsychological deficits 24 months after the hemorrhage, DCI seems to play an important role in the development of neuropsychological deficits in the chronic phase after aSAH.

### Neuropsychological outcome

The NAB was developed to evaluate neurologically impaired patients aged 18 to 97 [23]. In clinical practice, the NAB screening module has only been administered to small inhomogeneous cohorts of patients with mixed neurological pathologies such as traumatic brain injury or brain tumors [36]. To our knowledge, in the current study it was systematically administered to patients with aSAH for the first time. In our cohort the NAB-S could be administered to 75.9 and 88.9% of the patients at 12 and 24 months, respectively. Therefore, administration of the NAB-S at 12 and 24 months after aSAH is well feasible, however, as only 50.0% of the patients with high grade aSAH could be tested at 3 months, the battery is probably more useful for low grade aSAH patients at this timepoint.

### Return to work

Returning to work is a very important goal of rehabilitation and social reintegration for patients after suffering from aSAH; however, in our study population only a small number of patients who returned for follow up returned to work to their previous capacity (0, 23.5 and 37.5% at 3, 12 and 24 months, respectively) which is well in line with previous reports [30, 37, 38]. Therefore, predicting the ability to return to work is a very important requirement for a neuropsychological assessment tool to be of clinical use. In our study, no patient who returned to work had severe impairments of any or moderate impairments of two or more domains of the NAB-S. If a severe deficit was present at 12 or 24 months the patient was significantly less likely to return to work. Furthermore, the patient was also less likely to return to work if two or more domains were moderately impaired at 12 or 24 months. Therefore, the NAB-S performed very well in detecting neuropsychological deficits that were relevant for predicting a patient’s ability to return to work at previous capacity. This is of even more interest as Wallmark et al. reported that return to work could be predicted using the MoCA [37]. However, Wallmark et al. did not report data on MoCA scores and occupational status at 24 months and were able to correctly predict occupational status in only 68% of the cases. In our study, non-impaired MoCA scores of 26 points or more did not significantly correlate with a higher likelihood to return to work at any timepoint and there were patients who had returned to work to their previous capacity despite having scored 25 or less points on the MoCA at 12 as well as 24 months after the bleeding. Therefore, we feel that the MoCA test is not useful to detect relevant neuropsychiological deficits prohibiting patients from returning to work and that using the NAB-S generates important additional information on the patient’s neuropsychological status and ability to return to work compared to the MoCA.

### Neurological recovery following aSAH

In addition to neuropsychological assessments, the current study prospectively evaluated clinical outcome up to 24 months after aSAH. It could be shown that significant recovery assessed by GOSE which has been the most important outcome parameter in large clinical trials continues throughout the first 2 years after the bleeding. However, many large randomized clinical trials which failed to show any beneficial effects of various therapeutic strategies for aSAH evaluated outcome at 3 months only [4, 11–16]. Therefore, effects on long term outcome might have been missed due to too short follow up. Future studies should account for the fact that clinical recovery continues for at least 24 months after aSAH and include longer follow up schedules of at least 12 or if possible 24 months.

### Limitations

In the current study, due to the acute nature of aSAH no information on premorbid neurocognitive function can be presented, as extensively evaluating patients` neurocognitive function before an acute event is naturally not possible. However, this might affect evaluation of neuropsychological outcome at the follow-up visits as patients might have been impaired even before the acute event of aSAH. Furthermore, numbers of patients especially at later stages of follow-up are limited as we present our first single center experiences with the NAB-S in patients with aSAH. Due to this, exact analysis of temporal patterns of intraindividual functional and neuropsychological recovery, which would of course be of great interest, is significantly hampered. Finally, at 3 months after the hemorrhage, the NAB-S could not be administered to a third of the patients due to clinical condition; therefore, the use of the NAB-S in all aSAH patients at this timepoint cannot be generally recommended and might be more appropriate in low grade aSAH patients only.

## Conclusion

In the current study, the NAB screening module was used to neuropsychologically assess patients with aSAH for the first time. It could be administered to the majority of patients at 12 and 24 months after SAH; therefore, clinical use is feasible. At 3 months, the battery is probably more useful for low grade aSAH patients as at this timepoint only 50% of the high grade SAH patients could be tested. Neuropsychological deficits detected by the NAB-S were significantly correlated to patients` ability to return to work as well as clinical outcome assessed by GOSE; therefore, it provided valuable additional information on patients` functional status. Finally, it could be shown that recovery after aSAH continues for at least 24 months after the hemorrhage; therefore, in order to detect long term therapeutic effects future clinical studies investigating aSAH should assess clinical outcome for a longer period of time as it has been the case in the past.

## Data Availability

The datasets used and/or analysed during the current study are available from the corresponding author on reasonable request.

## Abbreviations

*aSAH*: Aneurysmatic subarachnoid hemorrhage
*CSF*: Cerebrospinal fluid
*CT*: Computed tomography
*DCI*: Delayed cerebral ischemia
*DSA*: Digital subtraction angiography
*GCS*: Glasgow Coma Scale
*GOSE*: Extended Glasgow Outcome Scale
*ICU*: Intensive care unit
*IMC*: Intermediate care unit
*mRS*: Modified Rankin Scale
*MoCA*: Montreal Cognitive Assessment
*MMSE*: Mini-Mental Status Evaluation
*NAB*: Neuropsychological Assessment Battery
*NAB-S*: Neuropsychological Assessment Battery Screening Module
*pTiO2*: Brain tissue partial oxygen pressure
*SAH*: Subarachnoid hemorrhage
*WFNS*: World Federation of Neurological Surgeons
